# Fizzy: feature subset selection for metagenomics

**DOI:** 10.1186/s12859-015-0793-8

**Published:** 2015-11-04

**Authors:** Gregory Ditzler, J. Calvin Morrison, Yemin Lan, Gail L. Rosen

**Affiliations:** 1 Department of Electrical & Computer Engineering, The University of Arizona, 1230 E Speedway Blvd., ECE Bldg., Tucson, 85721 AZ USA; 2 Department of Electrical & Computer Engineering, Drexel University, 3141 Chestnut St., Philadelphia, 19104 PA USA; 3 School of Biomedical Engineering, Science and Health, Drexel University, 3141 Chestnut St., Philadelphia, 19104 PA USA

**Keywords:** Feature subset selection, Comparative metagenomics, Open-source software

## Abstract

**Background:**

Some of the current software tools for comparative metagenomics provide ecologists with the ability to investigate and explore bacterial communities using *α*– & *β*–diversity. Feature subset selection – a sub-field of machine learning – can also provide a unique insight into the differences between metagenomic or 16S phenotypes. In particular, feature subset selection methods can obtain the operational taxonomic units (OTUs), or functional features, that have a high-level of influence on the condition being studied. For example, in a previous study we have used information-theoretic feature selection to understand the differences between protein family abundances that best discriminate between age groups in the human gut microbiome.

**Results:**

We have developed a new Python command line tool, which is compatible with the widely adopted BIOM format, for microbial ecologists that implements information-theoretic subset selection methods for biological data formats. We demonstrate the software tools capabilities on publicly available datasets.

**Conclusions:**

We have made the software implementation of Fizzy available to the public under the GNU GPL license. The standalone implementation can be found at http://github.com/EESI/Fizzy.

## Background

There is an immense amount of sequence data being collected from the next generation sequencers. Sequences from bacterial communities are collected from whole genome shotgun (WGS), or amplicon sequencing runs, and the analysis of such data allows researchers to study the functional or taxonomic composition of a sample. Microbial ecologists represent the composition in the form of an abundance matrix, which usually holds counts of operational taxonomic units (OTUs), but can also hold counts of genes/metabolic pathway occurrences if the data are collected from WGS. Furthermore, collections of metagenomic samples contains different factors, or phenotypes, such as environmental pH and salinity values, or a health related status [1, 2].

In this work, we introduce software tools for microbial ecologist researchers that implement feature subset selection routines for biological data formats. Prior to feature selection, we assume that the raw sequences from the environmental samples have already been classified into operational taxonomic units (OTUs), or functional features. The raw OTU counts are stored in a matrix $\mathbf {X} \in N_{+}^{K \times M}$, where *N*_+_ is the set of positive natural numbers, *K* is the number of OTU clusters, and *M* is the number of samples collected. The *M* samples contain a significant amount of *meta-data* describing the sample, which is where we obtain phenotypes describing the sample. While there may be many different meta-data, we shall only focus on one piece of meta-data at a time. For example, a sample may contain the sex, age, and height of the person from where a sample was collected, and the analysis would only use one of those fields. That is we could use **X** to build a predictive model of sex. Both the data matrix and meta-data can be found for hundreds of publicly available datasets through pioneering projects such as MG-RAST [3], KBase [4], the Human Microbiome Project [5], and the Earth Microbiome Project [6].

A natural question to ask about studies with multiple phenotypes is: “which OTUs or functions are important for differentiating the phenotypes?” Answering such a question can be useful for understanding which conditions are driving/being affected by differences in composition and function across samples. Subset selection is the process of taking a high-dimensional dataset and reducing the size of the feature set by allowing the reduced subset to contain only *relevant* features [7]. Subset selection can also produce a feature subset that not only removes irrelevant features (i.e., features that do not carry information about the phenotype), but also does not contain features that are redundant (i.e., features carry the same information). This process of reducing the feature set offers a rapid insight into uncovering the differences between multiple populations in a metagenomic study and can be performed as complementary analysis to *β*-diversity methods, such as PCoA. Feature selection has been performed previously, by tools such as Random Forests [8], and Lefse [9], but is usually tied to a classification type or effect size.

## Methods

### Information-theoretic subset selection

One of the fundamental quantities in information theory that has been widely adopted for feature subset selection with filters is *mutual information*, which is given by:

(1) $$\begin{array}{*{20}l} \textsf{I}(X;Y) = \sum_{y\in\mathcal{Y}}\sum_{x\in\mathcal{X}} p_{X,Y}(x,y) \log \frac{p_{X,Y}(x,y)}{p_{X}(x)\,p_{Y}(y)}  \end{array} $$

where *p*_*X*_(*x*) is the marginal distribution over the random variable *X*, and *p*_*X*,*Y*_(*x*,*y*) is the joint probability distribution over *X* and *Y*. The supports of the random variables *X* and *Y* are defined by $\mathcal {X}$ and $\mathcal {Y}$. The mutual information can be used as scoring function for determining the set of features $\mathcal {F}$ that carry the most information about an outcome *Y*.

**Fig. 1:**
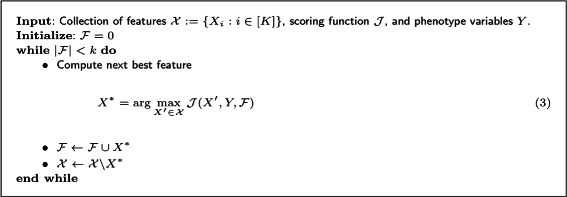
Pseudo code for search selecting features using a greedy algorithm that attempts to maximize $\mathcal {J}$

A simple algorithm for feature selection with a filter is a *greedy forward selection search* that seeks to maximize feature scoring function $\mathcal {J}$, which is shown in Fig. 1. The search initializes the relevant feature set $\mathcal {F}$ be empty, then for *k* iterations, an objective function $\mathcal {J}$ is maximized. For example, this objective function could be written as

(2) $$\begin{array}{*{20}l} {}\mathcal{J}(X, Y, \mathcal{F}) = \textsf{I}(X;Y) - \alpha\!\sum_{X'\in\mathcal{F}} \textsf{I}(X;X') + \beta\sum_{X'\in\mathcal{F}} \textsf{I}(X;X'|Y)  \end{array} $$

where *α*,*β*≥0. The first term in the expression captures the relevancy of the variable *X*. The next two terms measure the redundancy and conditional redundancy of *X* with the relevant feature set $\mathcal {F}$, respectively. Note that the sign of the conditional redundancy is positive to reward features being jointly informative about the class variable *Y*. The feature that maximizes this expression is added to the relevant feature set, $\mathcal {F}$, and removed from the feature set, $\mathcal {X}$. Simply using mutual information as the objective function is a fast way for microbial ecologists to examine the relative importance of taxa in a study collected from environmental samples. However, simply using mutual information will not capture inter-feature dependencies. Using other objective functions, such as joint mutual information [10] $\left (\alpha =\beta =\frac {1}{|\mathcal {F}|}\right)$, or mRMR [11] $\left (\alpha = \frac {1}{|\mathcal {F}|},\,\beta =0\right)$, captures some of the inter-feature dependencies.

Our recent work developed the Neyman-Pearson Feature Selection (NPFS), which automatically detects the relevant features in a dataset using a generic scoring function $\mathcal {J}$ [12, 13]. NPFS is highly parallelizable, which allows it to be quite effective for very large datasets. NPFS works by mapping out random samples of the original dataset to a scoring function which makes a prediction on which features are relevant. All of the sub-datasets have the same number of features selected then in a reduction phase NPFS applied the Neyman-Pearson test to detect feature importance. In this setting, NPFS can detect the number of important OTUs simply by guessing *k* in Fig. 1 for the scoring function and letting the hypothesis detect features that appear to be more important. NPFS was found to improve traditional methods of feature selection, while remaining highly parallelizable.

### Subset selection via regularization

Section ‘Information-theoretic subset selection’ introduced a greedy algorithm and tools from information theory that can be used to select features that are deemed important by the scoring function. Now we present feature selection from an embedded perspective. Let **y** be a vector in {±1}^*M*^ containing a binary outcome (e.g., control or stimulus) and **X** be abundance matrix. Predictions are made on **y** with **X** ^T^ *θ*, where *θ*∈*R* ^*K*^. If many of the entries of *θ* were zero then we could view the inner product of *θ* with **X** as a form of feature selection. To encourage sparsity in *θ*’s solution, Tibshirani presented lasso, which adds a penalty to the *l*_1_-norm of *θ* [14]. Formally, lasso is given by:

(3) $$\begin{array}{*{20}l} \theta^{*} = \arg\min_{\theta\in\Theta} \frac{1}{2M} \| \mathbf{y} - \mathbf{X}^{\textsf{T}}\theta \|_{2}^{2} + \lambda \|\theta\|_{1}  \end{array} $$

where *λ*>0, and ∥·∥_1_ and ∥·∥_2_ are the *l*_1_- and *l*_2_-norms, respectively. For lasso to be effective at feature selection, it is assumed that *K*≫*M*, which is typically an acceptable assumption with 16S and metagenomic data because there are typically only a few samples and a large number of features.

## Software implementations

Fizzy is a suite of subset selection tools that takes the Biom standard format [15] as input due to its acceptance into the standards by the Genomic Standards Consortium (http://gensc.org). Commonly used software for analyzing data from microbial ecology, such as Qiime [16], requires a Biom file containing the 16S data and a map file contain the meta-data of the samples within the Biom file. However, Fizzy allows users to store the meta-data in the Biom file directly, thus avoiding requirements for both a Biom and map file.

The Fizzy software suite implements information-theoretic subset selection, NPFS, and lasso. The core of Fizzy is based on the FEAST C feature selection library [17], which is used to implement all of the information theoretic methods. FEAST was selected for two primary reasons: (i) the library contains a large selection of information-theoretic feature selection objective functions, and (ii) the run-time of FEAST is typically faster than other feature selection libraries because it is written in a compiled language. We implemented a Python interface for FEAST to use within Fizzy, which is available to the public^1^. The Fizzy tool requires a Biom format OTU table (sparse or dense), a mapping file in tab-delimited (TSV) format, a metagenomic phenotype column in the map file, and an output file path be specified. Furthermore, Fizzy allows the user to specify the number of taxonomic units to select as well as the feature selection objective function. The current implementation of Fizzy has nine subset selection objective functions, which are all based on information theory (see Brown et al. for the mathematical details about the objective functions [17]). We also provide an implementation of the NPFS module, which can infer on the number of relevant features given any subset selection methods in FEAST [12]. Since NPFS works on top of a generic scoring function, we indicate the scoring function with NPFS as NPFS-SF, where SF is a scoring function such as MIM, mRMR or JMI. NPFS has a parallel implementation where the user can control the number of cores used by the program. The lasso implementation within Fizzy uses Scikit-Learn [18]. The regularization parameter for lasso is found using cross-validation and a grid search, where the values swept over the grid are determined from the data. The *λ* that minimizes the cost function is chosen as the final model.

## Benchmark data sets

We benchmarked Fizzy using data collected from the American Gut (AG) Project [19], and Qin et al.’s study of IBD patients [1] (both datasets are publicly available). The gut samples from the AG Project study are filtered into a separate Biom file for Fizzy and the diet type of the individual is the metagenomic phenotype. Diet was discriminated based on whether peoples’ diets included terrestrial animals, with Omnivores including those who ate chicken and/or red meat. Vegetarians included those who ate seafood, but no terrestrial animals. Qin et al.’s data are sampled from the gut and we use IBD and control as the metagenomic phenotype. The data used in our experiments have been made publicly available^2^.

## Results and discussion

We compared five algorithms on the American Gut Project data set: JMI (Table 1), NPFS-JMI (Table 2), Random Forest Classifiers (RFC) (Table 3), Lefse (Table 4), and lasso (no table due to only one feature selected – see below). The regularization parameter for lasso, *λ* in (3), was chosen to be 1.188×10^−3^ after performing cross validation. JMI was implemented in Fizzy, Lasso is available through our implementation, NPFS-JMI is our novel method, and these are compared to current popular methods such as RFC (used in [16, 20]) and Lefse.

**Table 1:** List of the top ranking features for omnivores and vegetarians in the 16S data collected from the American Gut Project detected using JMI within Fizzy

(Feature rank)	Operation taxonomic unit classification	(OTU ID)
(F1)	Firmicutes, Clostridia, Clostridiales, Lachnospiraceae	(GGID4329132)
(F2)	Firmicutes, Clostridia, Clostridiales, Ruminococcaceae	(GGID185584)
(F3)	Bacteroidetes, Bacteroidia, Bacteroidales, Bacteroidaceae, Bacteroides	(GGID177150)
(F4)	Bacteroidetes, Bacteroidia, Bacteroidales, Bacteroidaceae, Bacteroides	(GGID197367)
(F5)	Bacteroidetes, Bacteroidia, Bacteroidales, Bacteroidaceae, Bacteroides	(GGID199716)
(F6)	Bacteroidetes, Bacteroidia, Bacteroidales, Bacteroidaceae, Bacteroides	(GGID188887)
(F7)	Bacteroidetes, Bacteroidia, Bacteroidales, Bacteroidaceae, Bacteroides	(GGID312140)
(F8)	Bacteroidetes, Bacteroidia, Bacteroidales, Bacteroidaceae, Bacteroides	(GGID4401110)
(F9)	Bacteroidetes, Bacteroidia, Bacteroidales, Bacteroidaceae, Bacteroides	(GGID198449)
(F10)	Firmicutes, Bacilli, Bacillales, Paenibacillaceae, Paenibacillus	(GGID4470837)
(F11)	Firmicutes, Clostridia, Clostridiales, Ruminococcaceae, Faecalibacterium prausnitzii	(GGID359314)
(F12)	Bacteroidetes, Bacteroidia, Bacteroidales, Bacteroidaceae, Bacteroides	(GGID2859978)
(F13)	Firmicutes, Clostridia, Clostridiales	(GGID197832)
(F14)	Bacteroidetes, Bacteroidia, Bacteroidales, Bacteroidaceae, Bacteroides	(GGID205904)
(F15)	Firmicutes, Clostridia, Clostridiales, Ruminococcaceae, Faecalibacterium prausnitzii	(GGID520413)

The number followed by “F” indicates the order Fizzy selected the OTU and the “GGID” contains the Greengenes OTU ID from the taxonomic classification

**Table 2:** List of the top ranking features for omnivores and vegetarians in the 16S data collected from the American Gut Project detected using NPFS-JMI

(Feature rank)	Operation taxonomic unit classification	(OTU ID)
(F1)	Firmicutes, Clostridia, Clostridiales, Lachnospiraceae, Shuttleworthia	(GGID4424924)
(F2)	Cyanobacteria, Oscillatoriophycideae, Chroococcales, Xenococcaceae, Chroococcidiopsis	(GGID649518)
(F3)	Proteobacteria, Betaproteobacteria, Gallionellales, Gallionellaceae, Gallionella	(GGID3239358)
(F4)	Firmicutes, Clostridia, Clostridiales	(GGID176062)
(F5)	Firmicutes, Bacilli, Gemellales, Gemellaceae	(GGID967433)
(F6)	Firmicutes, Erysipelotrichi, Erysipelotrichales, Erysipelotrichaceae, Erysipelothrix	(GGID4478325)
(F7)	Firmicutes, Clostridia, Clostridiales, Lachnospiraceae	(GGID183576)
(F8)	Firmicutes, Clostridia, Clostridiales, Clostridiaceae, Clostridium	(GGID174688)
(F9)	Firmicutes, Clostridia, Clostridiales, Clostridiaceae	(GGID1137375)
(F10)	Firmicutes, Clostridia, Clostridiales, Lachnospiraceae, Blautia	(GGID305997)
(F11)	Firmicutes, Clostridia, Clostridiales, Lachnospiraceae	(GGID288682)
(F12)	Proteobacteria, Gammaproteobacteria, Pasteurellales, Pasteurellaceae, Haemophilus	(GGID995893)
(F13)	Bacteroidetes, Bacteroidia, Bacteroidales, Bacteroidaceae, Bacteroides	(GGID4450198)
(F14)	Firmicutes, Clostridia, Clostridiales	(GGID267502)
(F15)	Bacteroidetes, Bacteroidia, Bacteroidales, Bacteroidaceae, Bacteroides	(GGID531722)

The number followed by “F” indicates the order NPFS selected the OTU and the “GGID” contains the Greengenes OTU ID from the taxonomic classification

**Table 3:** List of the top ranking features for omnivores and vegetarians in the 16S data collected from the American Gut Project detected using Random Forests

(Feature rank)	Operation taxonomic unit classification	(OTU ID)
(F1)	Bacteroidetes, Bacteroidia, Bacteroidales, Bacteroidaceae, Bacteroides ovatus	(GGID180606)
(F2)	Bacteroidetes, Bacteroidia, Bacteroidales, Bacteroidaceae, Bacteroides fragilis	(GGID4386507)
(F3)	Firmicutes, Clostridia, Clostridiales, Lachnospiraceae, Roseburia	(GGID4335815)
(F4)	Actinobacteria, Actinobacteria, Actinomycetales, Corynebacteriaceae, Corynebacterium simulans	(GGID912997)
(F5)	Bacteroidetes, Bacteroidia, Bacteroidales, Rikenellaceae	(GGID175375)
(F6)	Firmicutes, Clostridia, Clostridiales, Lachnospiraceae	(GGID194112)
(F7)	Firmicutes, Clostridia, Clostridiales, Ruminococcaceae	(GGID189924)
(F8)	Bacteroidetes, Bacteroidia, Bacteroidales, Bacteroidaceae, Bacteroides	(GGID1105984)
(F9)	Bacteroidetes, Bacteroidia, Bacteroidales, Bacteroidaceae, Bacteroides	(GGID197367)
(F10)	Firmicutes, Clostridia, Clostridiales, Ruminococcaceae	(GGID174818)
(F11)	Firmicutes, Clostridia, Clostridiales, Ruminococcaceae	(GGID4324040)
(F12)	Firmicutes, Clostridia, Clostridiales, Ruminococcaceae	(GGID197204)
(F13)	Bacteroidetes, Bacteroidia, Bacteroidales, Bacteroidaceae, Bacteroides	(GGID1944498)
(F14)	Firmicutes, Clostridia, Clostridiales, Ruminococcaceae	(GGID196307)
(F15)	Firmicutes, Clostridia, Clostridiales, Ruminococcaceae, Ruminococcus flavefaciens	(GGID1122673)

The number followed by “F” indicates the order the Random Forest selected the OTU and the “GGID” contains the Greengenes OTU ID from the taxonomic classification

**Table 4:** List of the largest differences in abundance between omnivores and vegetarians in the 16S data collected from the American Gut Project using LefSe. Note that LefSe does not return the Greengenes IDs

Operation taxonomic unit classification
Bacteria, Actinobacteria, Actinobacteria, Actinomycetales, Actinomycetaceae, Actinobaculum
Bacteria, Actinobacteria, Actinobacteria, Actinomycetales, Micrococcaceae, Kocuria, rhizophila
Bacteria, Proteobacteria, Gammaproteobacteria, Xanthomonadales, Xanthomonadaceae, Dyella
Archaea, Euryarchaeota, Methanomicrobia, Methanosarcinales
Bacteria, Proteobacteria, Alphaproteobacteria, Rhizobiales, Bradyrhizobiaceae, Bradyrhizobium
Bacteria, Actinobacteria, Actinobacteria, Actinomycetales, Mycobacteriaceae, Mycobacterium, celatum
Bacteria, Actinobacteria, Actinobacteria, Bifidobacteriales, Bifidobacteriaceae, Alloscardovia
Bacteria, Actinobacteria, Actinobacteria, Actinomycetales, Mycobacteriaceae
Bacteria, Actinobacteria, Actinobacteria, Actinomycetales, Actinomycetaceae, Actinomyces, europaeus
Bacteria, Actinobacteria, Actinobacteria, Actinomycetales, Micromonosporaceae
Bacteria, Proteobacteria, Betaproteobacteria, Burkholderiales, Comamonadaceae, Paucibacter
Bacteria, Firmicutes, Bacilli, Bacillales, Bacillaceae, Bacillus, coagulans
Bacteria, Firmicutes, Bacilli, Bacillales, Bacillaceae, Bacillus, humi
Archaea, Euryarchaeota, Methanomicrobia, Methanosarcinales, Methanosarcinaceae, Methanosarcina, mazei
Archaea, Euryarchaeota, Methanomicrobia
Archaea, Euryarchaeota, Methanomicrobia, Methanosarcinales, Methanosarcinaceae
Bacteria, Bacteroidetes, Flavobacteriia, Flavobacteriales, Flavobacteriaceae, Capnocytophaga
Bacteria, Proteobacteria, Alphaproteobacteria, Rhodospirillales, Acetobacteraceae, Acetobacter
Bacteria, Actinobacteria, Actinobacteria, Actinomycetales, Nocardioidaceae, Nocardioides

The algorithms were run on 2.9k + samples collected from the AG Project and feature were selected using the diet type as the predictor variable. The diets are are broken down into omnivore and vegetarians, where subcategories of omnivore and vegetarians (e.g., omnivore but does not eat red meat) is simply categorized as omnivore. Table 1 shows the top ranking OTUs as selected for differentiate omnivores versus vegetarians in the AG Project data. Both *Bacteroides* and *Prevotella* were detected in the variable selected by Fizzy (note that *Prevotella* is not shown in the table because it was not ranked within the top 15 OTUs), which have been hypothesized as being important differentiators of diet [21]. This effect was also observed when we evaluated only vegans and omnivores. NPFS detected 27 OTUs of the *Prevotella* genus and the relative abundances were larger for the vegetarians when examining the largest differences, which coincides with results in the literature [22]. Differences between the JMI & NPFS-JMI OTU rankings, could be due to a large cluster of features that carry similar relevance, which when with the bootstrapping in NPFS could rank them in a different order.

We also compare Fizzy to Qiime’s random forests [8] because random forest within Qiime has become a commonly used benchmark in microbial ecology, as well as LefSe [9]. The top ranked features for random forests are found in Table 3. Similar to of feature selection approaches such as mRMR and JMI, a threshold for the number of features to select must be chosen in advance. We find some overlap between the results of Fizzy (using JMI) and the random forests. The *Bacteroides* genus was detected as relevant several times for both Fizzy and random forests. We find the *Bacteroides* has been found to be an indicator of diet [23–25]. However, Lefse returns different subsets of feature than the proposed methods or the random forests (see Table 4).

**Fig. 2:**
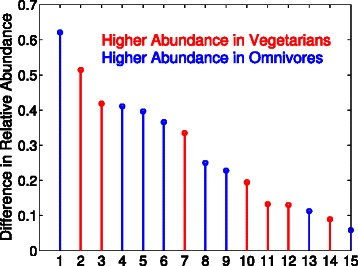
Joint Mutual Information (JMI) was configured to select 500 features from the 25k + OTUs in the American Gut Project’s fecal samples. The diet of the sample is the dependent variables. The selected Greengenes (GG) OTUs are sorted by the absolute difference between the omnivores and vegetarians. The numerical values on the x-axis that correspond to an OTU can be found the the text
(F4) *Bacteroidetes, Bacteroidia, Bacteroidales, Bacteroidaceae, Bacteroides* (GGID197367): 5.14587
(F127) *Firmicutes, Clostridia, Clostridiales, Lachnospiraceae* (GGID340761): 4.18384
(F223) *Firmicutes, Clostridia, Clostridiales, Ruminococcaceae* (GGID180285): –4.11038
(F291) *Bacteroidetes, Bacteroidia, Bacteroidales, Bacteroidaceae, Bacteroides ovatus* (GGID180606): –3.96605
(F206) *Firmicutes, Clostridia, Clostridiales, Ruminococcaceae* (GGID352347): –3.65923
(F195) *Bacteroidetes, Bacteroidia, Bacteroidales, Bacteroidaceae, Bacteroides* (GGID3465233): 3.34877
(F60) *Firmicutes, Clostridia, Clostridiales* (GGID173876): –2.49844
(F458) *Firmicutes, Clostridia, Clostridiales, Lachnospiraceae* (GGID193477): –2.28077
(F113) *Bacteroidetes, Bacteroidia, Bacteroidales, Rikenellaceae* (GGID4453609): 1.94571
(F463) *Firmicutes, Clostridia, Clostridiales, Lachnospiraceae, Ruminococcus gnavus* (GGID191755): 1.32321
(F310) *Bacteroidetes, Bacteroidia, Bacteroidales, Porphyromonadaceae, Parabacteroides* (GGID847228): 1.30030
(F276) *Firmicutes, Clostridia, Clostridiales, Lachnospiraceae, Coprococcus* (GGID2740950): –1.12856
(F257) *Bacteroidetes, Bacteroidia, Bacteroidales, Bacteroidaceae, Bacteroides* (GGID190913): 0.89408
(F106) *Firmicutes, Clostridia, Clostridiales, Lachnospiraceae* (GGID176306): –0.58509

Figure 2 shows the largest differences between the omnivores and vegetarians in the top 500 OTUs feature selected by JMI. The numerical values on the x-axis that correspond to the OTU given by:

(F148) *Bacteroidetes, Bacteroidia, Bacteroidales, Bacteroidaceae, Bacteroides uniformis* (GGID1733364): –6.20923

where the difference is ×10^−3^, (F#) is the order that JMI ranked the feature, GGID is the Greengenes ID, and a negative value means that the average relative abundance was higher in the vegetarians. Lasso selected only one OTU (*Ruminococcaceae*) after cross-validation, and a sweep of the regularization parameter, which increasing the regularization parameter could lead to more OTUs being selected at the cost of a larger error rate. It is interesting to observe that features 3 (F127) and 9 (F458) have opposing signs, yet the are the same family. We hypothesize that this result can be explained by different species will have different responses to environmental conditions. The top Pfams that maximize the mutual information for the MetaHit data set are shown in Table 5. It is known in IBD patients, the expression of ABC transporter protein (PF00005, the first feature MIM selected for classifying IBD vs. no IBD samples) is decreased which limits the protection against various luminal threats [26]. The feature selection for IBD also identified glycosyl transferase (PF00535), whose alternation is hypothesized to result in recruitment of bacteria to the gut mucosa and increased inflammation [27, 28], and the genotype of acetyltransferase (PF00583) plays an important role in the pathogenesis of IBD, which is useful in the diagnostics and treatment of IBD [29]. It is not surprising that ABC transporter (PF00005) is also selected for obesity, which is known to mediate fatty acid transport that is associated with obesity and insulin resistant states [30], and ATPases (PF02518) that catalyze dephosphorylation reactions to release energy.

**Table 5:** List of the top five ranked Pfams as selected by the Fizzy’s Mutual Information Maximization (MIM) applied to MetaHit

Rank	IBD features
*Feature 1*	ABC transporter (PF00005)
*Feature 2*	Phage integrase family (PF00589)
*Feature 3*	Glycosyl transferase family 2 (PF00535)
*Feature 4*	Acetyltransferase (GNAT) family (PF00583)
*Feature 5*	Helix-turn-helix (PF01381)
Rank	Obese features
*Feature 1*	ABC transporter (PF00005)
*Feature 2*	MatE (PF01554)
*Feature 3*	TonB dependent receptor (PF00593)
*Feature 4*	Histidine kinase-, DNA gyrase B-, and HSP90-like
	ATPase (PF02518)
*Feature 5*	Response regulator receiver domain (PF00072)

**Fig. 3:**
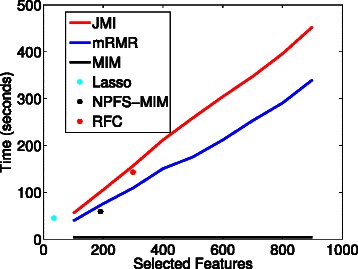
Number of feature being selected by JMI, mRMR, MIM Lasso, NPFS, and Random Forests as a function of the evaluation time

Figure 3 shows the evaluation time of six feature selection algorithms and the number of features they select evaluated on data collected from Caporaso et al. [31]. Both lasso and NPFS-MIM can select size of the relevant set, which is why they are represented as a single point. An interesting observation to make is that lasso selects very few features (nearly triple compared to NPFS-MIM). Though it should be noted lasso is capable of capturing more feature interdependencies than the current information theoretic approach presented in fizzy. Furthermore, Qiime’s RFC implementation is quite a bit slower than NPFS-MIM, but as with lasso, the RFC can capture large groups of feature interdependencies than the information-theoretic implementations. MIM, as expected, has the fast evaluation time because there is no calculation for redundancy, and the approaches that use redundancy (JMI and mRMR) take significantly longer to run. In fairness of comparison, the evaluation of NPFS can increase by choosing other base subset selection objective functions that incorporate a redundancy term.

## Conclusions

Feature subset selection provides an avenue for rapid insight to the taxonomic or functional differences that can be found between different metagenomic or 16S phenotypes in an environmental study. We have presented an information-theoretic feature subset selection, and lasso for biological data formats in Python that are compatible with those used with the software Qiime package. Furthermore, we have compared the results of our subset selection implementations on real-world 16S and metagenomic data, and we have compared our results to recent literature to ensure biological importance.

## Availability and requirements

**Project name**: Fizzy **Project home page**: https://github.com/EESI/Fizzy **Operating system(s)**: Linux and Mac OS X **Programming language**: Python and C **Other requirements**: Numpy^3^, PyFeast^4^ and Scikit Learn^5^ **License**: GNU GPL **Any restrictions to use by non-academics**: None

## Endnotes

^1^ http://github.com/EESI/PyFeast.

^2^ https://github.com/gditzler/DataCollections.

^3^ http://www.numpy.org/.

^4^ https://github.com/EESI/PyFeast.

^5^ http://scikit-learn.org/.

## References

[1] Qin J (2010). A human gut microbial gene catalogue established by metagenomic sequencing. Nature.

[2] Turnbaugh P (2009). A core gut microbiome in obese and lean twins. Nature.

[3] Meyer F, Paarmann D, D’Souza M, Olson R, Glass EM, Kubal M, et al. The metagenomics RAST server – a public resource for the automatic phylogenetic and functional analysis of metagenomes. BMC Bioinf.2008;9(386). http://www.biomedcentral.com/1471-2105/9/386. 10.1186/1471-2105-9-386 PMC2563014 18803844

[4] Department of Energy. DOE Systems Biology Knowledge Base. 2013. http://www.kbase.science.energy.gov.

[5] The NIH HMP Working Group (2009). The nih human microbiome project. Genome Res.

[6] Gilbert J (2010). Meeting Report: The Terabase Metagenomics Workshop and the Vision of an Earth Microbiome Project. Stand Genomic Sci..

[7] Guyon I, Elisseeff A (2003). An introduction to variable and feature selection. JMLR.

[8] Breiman L (2001). Random forest. Mach Lrn.

[9] Segata N, Izard J, Waldron L, Gevers D, Miropolsky L, Garrett WS (2011). Metagenomic biomarker discovery and explanation. Genome Biol..

[10] Yang H, Moody J. Data Visualization and Feature Selection: New Algorithms for Non-Gaussian Data. In: Advances in Neural Information Processing Systems: 1999.

[11] Peng H, Long F, Ding C (2005). Feature selection based on mutual information: criteria of max–dependency, max–relevance, and min–redundancy. IEEE Trans Pattern Anal Mach Intell.

[12] Ditzler G, Polikar R, Rosen G (2015). A bootstrap based neyman–pearson test for identifying variable importance. IEEE Trans Neural Netw and Learn Syst.

[13] Ditzler G, Austen M, Polikar R, Rosen G. Scaling a Subset Selection Approach Via Heuristics for Mining Massive Datasets. In: IEEE Symposium on Computational Intelligence and Data Mining: 2014. p. 439–45.

[14] Tibshirani R (1996). Regression shrinkage and selection via the lasso. J R Stat Soc.

[15] McDonald D, et al. The biological observation matrix (BIOM) format or: how I learned to stop worrying and love the ome-ome. GigaScience. 2012;1(7). http://www.ncbi.nlm.nih.gov/pmc/articles/PMC3626512/pdf/2047-217X-1-7.pdf. 10.1186/2047-217X-1-7 PMC3626512 23587224

[16] Caporaso JG, Kuczynski J, Stombaugh J, Bittinger K, Bushman FD, Costello EK (2010). QIIME allows analysis of high-throughput community sequencing data. Nat Methods.

[17] Brown G (2012). Conditional likelihood maximisation: a unifying framework for information theoretic feature selection. JMLR.

[18] Pedregosa F, Varoquaux G, Gramfort A, Michel V, Thirion B, Grisel O et (2011). Scikit-learn: Machine learning in Python. J Mach Learn Res..

[19] Knight R, Leach J, et al. The American Gut Project. Web. 2014.

[20] Kursa M, Rudnicki W. Feature selection with the boruta package. J Stat Softw.2010;36(11).

[21] Glick-Bauer M, Yeh MC (2014). The health advantage of a vegan diet: Exploring the gut microbiota connection. Nutrients.

[22] Wu G, Chen J, Hoffmann C, Bittinger K, Chen YY, Keilbaugh S (2011). Linking long-term dietary patterns with gut microbial enterotypes. Science.

[23] Matijašić B, Obermajer T, Lipoglavšek L, Grabnar I, Avguštin G, Rogelj I (2014). Association of dietary type with fecal microbiota in vegetarians and omnivores in slovenia. Eur J Nutr.

[24] Ruengsomwong S, Korenori Y, Sakamoto N, Wannissorn B, Nakayama J, Nitisinprasert S (2014). Senior thai fecal microbiota comparison between vegetarians and non-vegetarians using pcr-dgge and real-time pcr. J Microbiol Biotechnol.

[25] Kim MS, Hwang SS, Park EJ, Bae JW (2013). Strict vegetarian diet improves the risk factors associated with metabolic diseases by modulating gut microbiota and reducing intestinal inflammation. Environ Microbiol Rep.

[26] Deuring JJ, Peppelenbosch MP, Kuipers EJ, van der Woude CJ, de Haar C (2011). Impeded protein folding and function in active inflammatory bowel disease. Biochem Soc Trans.

[27] Theodoratou E (2014). The role of glycosylation in IBD. Nat Rev Gastroenterol Hepatol.

[28] Campbell B, Yu L, Rhodes J (2001). Altered glycosylation in inflammatory bowel disease: a possible role in cancer development. Glycoconj J.

[29] Baranska M, Trzcinski R, Dziki A, Rychlik-Sych M, Dudarewicz M, Skretkowicz J (2011). The role of n-acetyltransferase 2 polymorphism in the etiopathogenesis of inflammatory bowel disease. Dig Dis Sci.

[30] Ashrafi K. Obesity and the Regulation of Fat Metabolism: Worm Book; 2007, pp. 1–20. http://www.ncbi.nlm.nih.gov/pubmed/18050496. 10.1895/wormbook.1.130.1 PMC4780880 18050496

[31] Caporaso JG (2011). Moving pictures of the human microbiome. Genome Biol.

